# Trauma-related altered states of consciousness in women with BPD with or without co-occurring PTSD

**DOI:** 10.3402/ejpt.v5.24863

**Published:** 2014-08-18

**Authors:** Paul Frewen, Nikolaus Kleindienst, Ruth Lanius, Christian Schmahl

**Affiliations:** 1 Department of Psychiatry, Western University, London, ON, Canada; 2 Department of Psychology, Western University, London, ON, Canada; 3 Graduate Program in Neuroscience, Western University, London, ON, Canada; 4 Department of Psychosomatic Medicine, Central Institute of Mental Health, Medical Faculty Mannheim/ Heidelberg University, Mannheim, Germany

**Keywords:** Dissociation, borderline personality disorder, posttraumatic stress disorder, trauma-related altered states of consciousness, childhood abuse and neglect, 4-D model

## Abstract

**Background:**

A “4-D model” was recently described as a theoretical framework for categorizing trauma-related symptoms into four phenomenological dimensions (the experience of time, thought, body, and emotion) that can present either in the form of normal waking consciousness (NWC) or as dissociative experiences, that is, trauma-related altered states of consciousness (TRASC).

**Methods:**

The present study examined the predictions of the 4-D model in 258 persons with borderline personality disorder (BPD) with (*n*=126) versus without (*n*=132) posttraumatic stress disorder (PTSD).

**Results:**

As measured by the *Borderline Symptom List*, consistent with the predictions of the 4-D model, in comparison with symptom endorsements theorized to be associated with NWC, measures of TRASC were less frequent, and more strongly correlated with both *Dissociative Experience Scale* scores and severity of childhood emotional neglect, particularly in persons with both BPD and PTSD. Our prediction that symptoms of TRASC would be less intercorrelated in comparison with distress associated with NWC symptoms, however, was not supported.

**Conclusions:**

Findings are discussed as they pertain to the symptomatology of BPD, PTSD, and dissociation.

Borderline personality disorder (BPD) and posttraumatic stress disorder (PTSD) as defined by the fourth edition of the *Diagnostic and Statistical Manual* (DSM) are frequently comorbid psychiatric conditions. Studies show that between 35 and 55% of BPD patients suffer from comorbid PTSD (Zanarini et al., 1998; Zimmermann, 1999) and, compared with patients with other personality disorders, BPD patients are approximately twice as likely to have a co-occurring diagnosis of PTSD (Golier et al., 2003). Moreover, attending to the presence of PTSD comorbidity within BPD samples is clinically significant given that tendencies toward self-harm and suicidality may be increased in individuals with comorbid PTSD relative to BPD alone (Cougle, Keough, Riccardi, & Sachs-Ericsson, 2009; Harned, Rizvi, & Linehan, 2010; Nepon, Belik, Bolton, & Sareen, 2010; Pagura et al., 2010). In addition, in one study, the presence of PTSD was shown to decrease the chance for remission of BPD 2.5-fold (Zanarini, Frankenburg, Hennen, Reich, & Silk, 2004; Zanarini et al., 2011).

Shared etiology and overlapping clinical features or mechanisms are among the probable causes for the high comorbidity rates for PTSD frequently observed in BPD samples. Referring to etiology, a risk factor for both BPD and PTSD includes childhood maltreatment history. For example, the prevalence of childhood sexual abuse in BPD patients ranges between 52 and 71% (Ball & Links, 2009; Zanarini, 1997), and Cutajar et al. (2010) found that the risk for BPD was increased about sixfold in persons with a history of childhood sexual abuse.

Referring to clinical features or mechanisms, various forms of affect dysregulation are common to both BPD and PTSD, with an increasing number of investigations seeking to discern the clinical significance of dissociative symptoms variably present in both conditions. Within BPD patients, dissociation is often state-dependent and closely related to stress levels (Ludäscher, Bohus et al., 2007; Stiglmayr et al., 2008). Research has also shown that dissociative experiences in BPD patients interfere with pain sensitivity (Ludäscher, Bohus et al., 2007; Ludäscher, Valerius et al., 2010), amygdala, insula, and ACC response during emotional distraction occurring in the context of working memory performance (Krause-Utz et al., 2012) as well as the capacity for fear conditioning (Ebner-Priemer et al., 2009) in experimental paradigms. In addition, dissociative experiences at baseline showed a strong negative correlation with improvement after a 3-month DBT treatment even after controlling for overall baseline BPD symptom severity (Kleindienst et al., 2011).

In comparison, a dissociative subtype of PTSD, referring specifically to symptoms of derealization and depersonalization, was recently recognized to occur in approximately 15–30% of persons with PTSD (Armour, Karstoft, & Richardson, 2014; Stein et al., 2013; Steuwe, Lanius, & Frewen, 2012; Wolf, Lunney, et al., 2012; Wolf, Miller, et al., 2012). Within PTSD patients, such dissociative experiences were negatively correlated with arousal and positively correlated with medial prefrontal response during neuroimaging studies of emotional processing, including response to personal trauma reminders (reviewed by Lanius et al., 2010; Lanius, Brand, Vermetten, Frewen, & Spiegel, 2012; Lanius et al., 2014). Studies of the outcomes of psychological treatment, however, have largely failed to identify dissociative symptoms as a hypothesized negative prognostic predictor (Cloitre, Petkova, Wang, & Lu, 2012; Hagenaars, van Minnen, & Hoogduin, 2010; Halvorsen, Stenmark, Neuner, & Nordahl, 2014; Resick, Suvak, Johnides, Mitchell, & Iverson, 2012). However, Price, Kearns, Houry, and Rothbaum (2014) point out that “a potential explanation for the discrepancy between the theorized and empirical findings is the assessment of dissociation. [Previous] studies … assessed dissociation over the past week or month, whereas dissociation at the time of treatment is posited to reduce response” (Price et al., 2014, p. 2). Accordingly, Price et al. assessed dissociative symptoms present at an initial treatment session for PTSD in persons presenting at emergency departments following acute traumatic events, with 41% having experienced a recent sexual assault, and many reporting a prior history of childhood maltreatment. Although Price et al. used only a brief (3-session) prolonged exposure treatment, rendering comparisons with standard-length interventions (i.e., typically 8-session treatments) more difficult, they found that dissociative symptoms were the only significant predictor of reduced treatment response at 1 and 3 months post incident.

As a means of better understanding symptoms of dissociation not only in persons with PTSD but transdiagnostically across trauma-related disorders, Frewen and Lanius (in press) recently proposed a four-dimensional framework (the “4-D model”) that theoretically distinguishes dissociative experiences of trauma-related altered states of consciousness (TRASC) from what they consider to be non-dissociative forms of distress, that is, those symptoms of distress that are frequently experienced within normal waking consciousness (NWC). The four dimensions to which the 4-D model refers are the consciousness of: 1) time, 2) thought, 3) body, and 4) emotion. In considering the following as forms of TRASC, the 4-D model differentiates: 1) posttraumatic *flashbacks* from other forms of intrusive recall and reminder distress that do not entail a prominent experience of reliving (*time* dimension); 2) *voice-hearing* from negative self-referential thoughts that are experienced in first-person perspective (*thought* dimension); 3) disembodied experiences of *depersonalization* from embodied experiences of distress (*body* dimension); and 4) experiences of emotional *numbing* and affective *shut-down* from non-dissociative forms of negative emotionality (e.g., experiences of fear, anxiety, sadness, guilt, or shame; *emotion* dimension). The 4-D model further hypothesizes that experiences of TRASC, when compared with forms of general distress that remain part and parcel to NWC, will: 1) be observed less frequently (due to NWC being, by definition, the expected baseline phenomenological state of human beings, and previous studies showing that most persons with PTSD are *not* diagnosed with the dissociative subtype; Amour et al., 2014; Stein et al., 2013; Steuwe et al., 2012; Wolf et al., 2012a, b); 2) be less intercorrelated, particularly when measured as moment-to-moment states, viewing the four dimensions as more “compartmentalized” [Brown, 2006; Holmes et al., 2005] when expressed as TRASC); 3) be more strongly correlated with other measures of dissociative experience, for example, the *Dissociative Experiences Scale* (Bernstein & Putnam, 1986); and 4) be observed more specifically in chronically and developmentally traumatized persons in comparison with less repeatedly traumatized populations, because of hypothesized deleterious effects of early traumatization on the development of reality orientation (e.g., Carrick, Quas, & Lyon, 2010) and sense of self (Cicchetti & Lynch, 1993; Cole & Putnam, 1992).

Studies conducted by Frewen and Lanius (2014) recently evaluated the aforementioned four hypotheses of the 4-D model in women with PTSD related to childhood trauma and an undergraduate sample, respectively. Both studies evaluated and supported the first two hypotheses referring to lower frequency and intercorrelation of symptom endorsements of TRASC relative to NWC general distress dimensions. Moreover, the PTSD study also supported the third hypothesis regarding higher correlations of TRASC with other indicators of dissociation (this hypothesis was not tested within the undergraduate study). However, the fourth hypothesis concerning repeated and developmental trauma was not well supported in the PTSD sample (again untested within the undergraduate sample). Specifically, it was found that only severity of childhood sexual abuse, but not other forms of childhood maltreatment history, predicted experiences of voice-hearing in women with PTSD; dimensions of TRASC and distress associated with normal waking consciousness (NWC-distress) were unrelated with other types of self-reported childhood trauma history (i.e., physical or emotional abuse and neglect). Comorbidity of BPD was not investigated, however, and further transdiagnostic investigations of the 4-D model in individuals characterized by varying levels of childhood trauma exposure and dissociative symptoms are needed (Frewen & Lanius, 2014).

The goal of the present study was to examine BPD and PTSD comorbidity as a function of childhood trauma history and dissociative symptomatology by further evaluating predictions of the 4-D model in a sample of 295 women with BPD who differed from each other as a function of PTSD comorbidity, severity of childhood trauma history, and extent of dissociative symptoms. Following the 4-D model, we hypothesized that BPD symptoms reflective of TRASC, when compared with BPD symptoms associated with NWC, would be: 1) endorsed less frequently, 2) less intercorrelated, 3) more strongly correlated with dissociative experiences measured independently, and 4) more strongly correlated with childhood trauma histories. In addition, we evaluated whether results depended on comorbidity with PTSD. In particular, we predicted that symptoms of TRASC would be endorsed more frequently by individuals with BPD comorbid with PTSD, even after controlling for symptoms of NWC-distress.

## Method

### Participants

Inclusion criteria (4) for this study were: 1) female gender, 2) age 18–55, 3) German speaking, and 4) current primary diagnosis of BPD as determined by the *International Personality Disorder Examination* and assessed further by the *Borderline Symptom List* (BSL; Bohus et al., 2007). Exclusion criteria (3) for the study were: 1) presence of past PTSD in the absence of current PTSD; 2) Bipolar I or any current psychotic disorder and lifetime diagnosis of Schizophrenia, Schizophreniform, or Schizoaffective Disorder; and 3) psychiatric problems secondary to traumatic brain injury. Four hundred and twenty-seven (*n=*427) individuals were assessed for study inclusion criteria within the context of assessment for suitability for a BPD treatment offered to women between April 2001 and October 2007 at the Department of Psychiatry of the University in Freiburg, and at the Central Institute of Mental Health in Mannheim, Germany. Of these, 258 women (*n*=258) aged 18–55 (*M*=28.41, *SD*=7.76) met study inclusion criteria (the majority of participants were excluded due to non-primary diagnosis of BPD, failure to complete the BSL, and presence of past PTSD in the absence of current PTSD). Of the study sample of 258, 126 (*n*=126) participants were further diagnosed with current PTSD following the Structured Clinical Interview for DSM-IV-TR (SCID-I) whereas for the remaining 132 participants current PTSD was determined to be absent; such groups did not differ by age, *t*(256)=0.36, *p*=0.97. Table 1 reports demographic information, comorbid diagnoses, and other clinical indicators separately for participants with versus without PTSD.

**Table 1:** Sample clinical characteristics

	BPD with PTSD (*n*=126)	BPD without PTSD (*n*=132)	Difference between groups
Age	28.43 (8.08)	28.39 (7.48)	*t*(256)=0.36, *p=*0.97
BSL total	215.12 (63.23)	201.92 (70.10)	*t*(253)=1.58, *p=*0.12
BSL TRASC	1.62 (0.79)	1.35 (0.91)	*t*(256)=2.49, *p=*0.01
BSL NWC-distress	2.39 (0.79)	2.28 (0.90)	*t*(256)=1.40, *p=*0.16
DES	33.17 (16.59)	27.40 (14.59)	*t*(210)=2.69, *p=*0.01
CTQ – Emotional abuse	18.73 (5.32)	16.85 (5.26)	*t*(221)=2.65, *p=*0.01)
CTQ – Physical abuse	12.46 (6.40)	8.83 (4.78)	*t*(222)=4.84, *p<*0.001
CTQ – Sexual abuse	15.35 (7.73)	8.19 (4.97)	*t*(212)=8.17, *p<*0.001
CTQ – Emotional neglect	18.41 (4.86)	17.04 (4.87)	*t*(218)=2.07, *p=*0.04
CTQ – Physical neglect	10.73 (4.13)	9.56 (3.84)	*t*(222)=2.20, *p=*0.03
SCID-I comorbidity			
Bipolar disorder I	0 (L) 0 (C)	0 (L) 0 (C)	χ^2^=0 χ^2^=0
Bipolar disorder II	5 (L) 2 (C)	2 (L) 1 (C)	χ^2^=1.50, *p*=0.22 χ^2^=0.39, *p*=0.53
Other bipolar disorder	3 (L) 3 (C)	0 (L) 0 (C)	χ^2^=3.11, *p*=0.08 χ^2^=3.21, *p*=0.07
Major depression	107 (L) 52 (C)	103 (L) 56 (C)	χ^2^=1.95, *p*=0.16 χ^2^=1.02, *p*=0.60
Dysthymia	28 (C)	32 (C)	χ^2^=0.17, *p*=0.68
Delusional disorder	0 (L) 0 (C)	1 (L) 0 (C)	χ^2^=0.96, *p*=0.33 χ^2^=0
Brief psychotic disorder	3 (L) 1 (C)	1 (L) 0 (C)	χ^2^=1.08, *p*=0.30 χ^2^=1.07, *p*=0.30
Substance-induced psychotic disorder	3 (L) 0 (C)	2 (L) 0 (C)	χ^2^=0.26, *p*=0.61 χ^2^=0
Psychotic disorder NOS	5 (L) 5 (C)	2 (L) 1 (C)	χ^2^=1.45, *p*=0.23 χ^2^=3.04, *p*=0.08
Substance abuse/dependence			
Alcohol	27 (L) 12 (C)	54 (L) 13 (C)	χ^2^=4.14, *p*=0.13 χ^2^=0.21, *p*=0.88
Sedatives	28 (L) 8 (C)	17 (L) 4 (C)	χ^2^=4.32, *p*=0.12 χ^2^=1.92, *p*=0.17
Cannabis	13 (L) 9 (C)	22 (L) 6 (C)	χ^2^=0.17, *p*=0.92 χ^2^=0.79, *p*=0.38
Stimulants	4 (L) 3 (C)	10 (L) 2 (C)	χ^2^=4.97, *p*=0.08 χ^2^=0.29, *p*=0.59
Opiates	3 (L) 0 (C)	4 (L) 0 (C)	χ^2^=3.77, *p*=0.15 χ^2^=0
Cocaine	3 (L) 0 (C)	9 (L) 0 (C)	χ^2^=2.95, *p*=0.23 χ^2^=0
Hallucinogens	4 (L) 1 (C)	5 (L) 0 (C)	χ^2^=0.15, *p*=0.93 χ^2^=1.04, *p*=0.31
Polysubstance	10 (L) 3 (C)	8 (L) 2 (C)	χ^2^=4.76, *p*=0.09 χ^2^=0.26, *p*=0.61
Other addictions	8 (L) 2 (C)	5 (L) 2 (C)	χ^2^=1.38, *p*=0.50 χ^2^=0
Panic disorder (PD)	39 (L) 36 (C)	32 (L) 24 (C)	χ^2^=1.69, *p*=0.19 χ^2^=3.95, *p<*0.05
PD with agoraphobia	28 (C)	17 (C)	χ^2^=2.84, *p*=0.09
Agoraphobia without PD	21 (L) 20 (C)	12 (L) 9 (C)	χ^2^=3.67, *p*=0.06 χ^2^=5.77, *p*=0.02
Social phobia	72 (L) 65 (C)	51 (L) 51 (C)	χ^2^=7.42, *p*=0.01 χ^2^=5.35, *p*=0.02
Specific phobia	29 (L) 29 (C)	18 (L) 17 (C)	χ^2^=3.99, *p*<0.05 χ^2^=4.97, *p*=0.03
OCD	29 (L) 27 (C)	21 (L) 15 (C)	χ^2^=2.39, *p*=0.12 χ^2^=5.21, *p*=0.02
GAD	13 (C)	10 (C)	χ^2^=0.73, *p*=0.39
Somatization disorder	10 (C)	5 (C)	χ^2^=2.08, *p*=0.15
Pain disorder	11 (C)	7 (C)	χ^2^=1.21, *p*=0.27
Non-specific somatoform disorder	2 (C)	1 (C)	χ^2^=0.39, *p*=0.54
Hypochondriasis	5 (C)	4 (C)	χ^2^=0.17, *p*=0.68
Body dysmorphic disorder	4 (C)	7 (C)	χ^2^=0.74, *p*=0.39
Anorexia Nervosa	29 (L) 9 (C)	26 (L) 5 (C)	χ^2^=0.21, *p*=0.65 χ^2^=0.54, *p*=0.46
Bulimia	39 (L) 23 (C)	41 (L) 22 (C)	χ^2^=0.05, *p*=.94 χ^2^=0.16, *p*=0.69
Binge eating disorder	22 (L) 20 (C)	16 (L) 12 (C)	χ^2^=1.49, *p*=0.22 χ^2^=2.62, *p*=0.11
Other DSM disorder	3 (L) 3 (C)	4 (L) 4 (C)	χ^2^=0.20, *p*=0.88 χ^2^=0.21, *p*=0.89

All *p*-values are two-tailed. Differences in *df* due to missing data. (L) refers to lifetime diagnosis, (C) refers to Current Diagnosis. For simplicity of presentation, frequencies are reported for comorbid substance abuse *or* dependence, however associated χ^2^ values reflect the distinction between substance abuse versus dependence (i.e., 3 categories were analyzed: 1) absent, 2) present-abuse, 3) present-dependence). SCID-I=Structured Clinical Interview for DSM-IV-TR, Axis 1 conditions; NOS=not otherwise specified; OCD=obsessive compulsive disorder; GAD=generalized anxiety disorder; DSM=diagnostic and statistical manual.

### Measures

#### Borderline Symptom List

The BSL is a 95-item self-rating instrument to quantify borderline typical symptomatology on seven subscales (self-image, affect regulation, self-destruction, dysphoria, loneliness, intrusion, and hostility). It is based on the DSM-IV criteria for BPD and the Diagnostic Interview for BPD—Revised Version (DIB-R). Severity of symptoms is self-rated on a 5-point Likert scale (“not at all” to “very strong”). Authors PF, RL, and CS selected BSL items that they unanimously agreed best matched the descriptions of posttraumatic TRASC and NWC-distress as described by the 4-D model (Frewen & Lanius, in press). The following 12 BSL items were classified as examples of TRASC referring to the 4-D model dimensions of consciousness of time, thought, body, and emotion: 16, 32, 36, 43, 54, 57, 61, 66, 67, 71, 81, and 92. In comparison, the following 23 BSL items were classified as examples of NWC-distress referring to the same four dimensions: 08, 04, 11, 14, 15, 20, 28, 30, 35, 40, 41, 44, 47, 49, 56, 60, 76, 77, 83, 88, 91, 93, and 94. Table 2 presents examples of BSL items that were categorized as TRASC versus NWC-distress according to the 4-D model dimensions of: 1) time, 2) thought, 3) body, and 4) emotion (items are presented in their original German form as well as previously published English translations). Beyond face validity, the subscales demonstrated excellent internal consistency (Cronbach's alpha) of 0.89 (TRASC 12 items) and 0.96 (NWC-distress 23 items).

**Table 2:** Example items from BSL descriptive of TRASC versus general distress associated with NWC

	TRASC	NWC
Time-memory	Item #32: “I had images that I was very much afraid of” (tauchten in mir Szenen auf, die mich stark ängstigten), Item #57: “I was tortured by images” (wurde ich von inneren Bildern gequält)	Item #41: “I could hardly control my memories” (konnte ich meine Erinnerungen kaum steuern)
Thought	Item #67: “I suffered from voices and noises from inside my head” (litt ich unter der Wahrnehmung von Stimmen oder Geräuschen von innen), Item #81: “I felt as if I had different people inside of me” (hatte ich den Eindruck, als ob es unterschiedliche Personen in mir gäbe)	Item #28: “I didn't believe in my right to live” (glaubte ich, keine Lebensberechtigung zu haben), Item #35: “I hated myself” (haßte ich mich selbst)
Body	Item #36: “I experienced parts of my body dissolving” (hatte ich das Gefühl, daß sich Teile meines Körpers auflösen), Item #43: “I couldn't feel parts of my body” (konnte ich Teile meines Körpers nicht spüren)	Item #8: “Everything felt tight inside of me” (zog sich in mir alles zusammen), Item #30: “I experienced stressful inner tension” (stand ich innerlich unter Hochspannung)
Emotion	Item #16: “I felt paralyzed” (erlebte ich mich wie erstarrt), Item #92: “I felt numb” (erlebte ich mich wie taub)	Item #4: “I was suffering from massive states of anxiety” (litt ich unter massiven Angstzuständen), Item #77: “I was full of despair” (war ich verzweifelt)

TRASC=trauma-related altered states of consciousness; NWC=normal waking consciousness.

#### Dissociative Experiences Scale

The Dissociative Experiences Scale (DES) (Bernstein & Putnam, 1986) is a widely recognized and used 28-item measure of dissociative experiences.

#### Childhood Trauma Questionnaire—short form

The Childhood Trauma Questionnaire—short form (CTQ; Bernstein & Fink, 1998; Bernstein et al., 2003) is a widely used and population standardized retrospective self-report survey of the extent to which a person experienced abuse and/or neglect during childhood and adolescence. The CTQ has five subscales (5-items per subscale): *emotional neglect*, *emotional abuse*, *sexual abuse*, *physical abuse*, and *physical neglect*.

### Procedure

Diagnostic interviews and questionnaires were administered by experienced psychologists within the context of a psychological treatment program for BPD at the Department of Psychiatry, University of Freiburg and at the Central Institute of Mental Health, Mannheim. The present research received ethical approval by the institutional review boards of the University of Freiburg as well as Heidelberg University.

### Statistical analysis

Referring to correction for multiple comparisons, the four hypotheses of the 4-D model, that symptoms of TRASC, when compared with BPD symptoms associated with NWC, would be: 1) endorsed less frequently, 2) less intercorrelated, 3) more strongly correlated with dissociative experiences measured independently, and 4) more strongly correlated with childhood trauma histories, were treated as independent families of tests. Each family of tests was first evaluated within the sample at large with an uncorrected threshold for statistical significance of *p*<0.01. In cases where statistical significance was observed, follow-up subgroup analyses referring to the presence-versus-absence of PTSD comorbidity were undertaken with uncorrected *p*<0.05 as the criterion for significance. We also evaluated whether symptoms of TRASC would be endorsed more frequently by individuals with BPD comorbid with PTSD versus those without PTSD, with *p<*0.05 as the criterion for statistical significance.

## Results

### Group comparisons

Table 1 shows descriptive statistics and the statistical significance of group comparisons referring to BSL item endorsement separately for BPD participants with versus without PTSD; diagnostic information is also included. BPD participants with versus without comorbid PTSD did not differ by age and, with a liberal threshold for significance (uncorrected *p<*0.05), differed by frequency of comorbid diagnoses only in the case of current panic disorder, current agoraphobia without panic disorder, lifetime and current social phobia, lifetime and current specific phobia, and current OCD, in which in all cases the comorbid PTSD group evidenced a higher comorbidity rate.

Participants with comorbid PTSD scored higher on BSL TRASC items, but not on BSL items indicative of NWC-distress. As predicted, symptoms of TRASC were endorsed more frequently by individuals with BPD comorbid with PTSD, even after controlling for symptoms of NWC-distress, *F*(1,255)=5.11, *p=*0.03, η^2^=0.02. Participants with comorbid PTSD also scored higher on the DES and on all CTQ measures of childhood trauma history (Table 1).

### Hypothesis 1: symptoms of TRASC will be endorsed less frequently than NWC symptoms

Within the full sample, supporting hypothesis 1, items from BSL TRASC, *M*=1.48, *SD*=0.86, were endorsed less frequently than were BSL items considered indicative of NWC-distress, *M*=2.35, *SD*=0.85, *t*(257)=25.73, *p*<0.001. This was also true when those with and without comorbid PTSD were examined separately: *t*(126)=16.33, *p<*0.001, and *t*(131)=20.28, *p<*0.001, respectively (Table 1).

### Hypothesis 2: symptoms of TRASC will be less intercorrelated than NWC symptoms

Correlations between endorsement rates for particular BSL TRASC items were calculated, as were correlations between endorsement rates for particular BSL NWC-distress items. Inconsistent with predictions, BSL TRASC items were *not* appreciably less intercorrelated (*M*_*r*_=0.37, *SD*_*r*_=0.14, Range: 0.10≤ *r*≤0.73) relative to BSL items classified as NWC-distress (*M*_*r*_=0.39, *SD*_*r*_=0.13, Range: 0.09≤ *r*≤0.82). This was neither found to be a strong effect in subsamples with comorbid PTSD (TRASC items: *M*_*r*_=0.31, *SD*_*r*_=0.17, Range: −0.02≤ *r*≤0.67; NWC items: *M*_*r*_=0.37, *SD*_*r*_=0.13, Range: 0.06≤ *r*≤0.84) or those without PTSD (TRASC items: *M*_*r*_=0.41, *SD*_*r*_=0.14, Range: 0.13≤ *r*≤0.76; NWC-distress items: *M*_*r*_=0.41, *SD*_*r*_=0.15, Range: 0.04≤ *r*≤0.84).

### Hypothesis 3: symptoms of TRASC will be endorsed more often by persons high in other measures of dissociation

Table 3 reports correlation statistics associating DES scores with each of BSL TRASC and BSL NWC-distress items. Consistent with predictions, within the full sample, DES scores were more strongly correlated (*p*<0.001) with endorsement of BSL TRASC items than they were with endorsement of BSL items characteristic of NWC-distress. Whereas this comparison remained significant (*p*=0.002) within the subsample without PTSD, the comparison failed to reach statistical significance (*p*=0.093) within the subsample with PTSD.

**Table 3:** Correlations between BSL items indicative of TRASC versus general distress associated with NWC and both DES and CTQ

		DES	EA	PA	SA	EN	PN
Full sample	TRASC	0.61**	0.16**	0.10*	0.23**	0.12*	0.17**
	NWC	0.48**	0.15*	0.06	0.16**	0.03	0.10
	Z	3.44**	0.26	0.96	1.68	2.02*	1.53
PTSD+	TRASC	0.60**	0.38**	0.12	0.24**	0.30**	0.21*
	NWC	0.51**	0.28**	0.10	0.14	0.14	0.11
	Z	1.66	1.53	0.24	1.39	2.18*	1.51
PTSD−	TRASC	0.60**	−0.04	0.00	0.16*	−0.04	0.10
	NWC	0.44**	0.03	−0.03	0.15	−0.07	0.08
	Z	3.25**	1.30	0.63	0.16	0.63	0.39

* *p<*0.05,

** *p<*0.01. Z statistic based on Meng et al. (1992). TRASC=trauma-related altered states of consciousness; NWC=normal waking consciousness; DES=Dissociative Experiences Scale; EA=emotional abuse; PA=physical abuse; SA=sexual abuse; EN=emotional neglect; PN=physical neglect; CTQ=Childhood Trauma Questionnaire. Childhood maltreatment history was measured retrospectively by self-report using the CTQ.

### Hypothesis 4: endorsement of symptoms of TRASC will be greatest in persons with more extensive histories of childhood abuse

Table 3 also reports correlation statistics associating CTQ scores with each of BSL TRASC and BSL NWC-distress items. Across the full sample, correlations between CTQ subscale scores and BSL TRASC items ranged between 0.10 and 0.23 (*M*_*r*_=0.16, *SD*_*r*_=0.05), whereas correlations between CTQ subscale scores and BSL NWC-distress items were generally lower, ranging between 0.03 and 0.16 (*M*_*r*_=0.10, *SD*_*r*_=0.06). The correlation between CTQ scores and TRASC was significantly stronger than the correlation between CTQ scores and NWC-distress items, however, only in the case of histories of emotional neglect (*p<*0.05). This generally remained the case when persons with comorbid PTSD were examined separately. However, within persons without PTSD comorbidity, CTQ measures were generally not significantly correlated with either BSL measures of TRASC or NWC-distress.

## Discussion

The aim of the present study was to examine experiences of TRASC, in comparison with those of general NWC-distress, following the predictions of the 4-D model of trauma-related dissociation (Frewen & Lanius, 2014, in press), in persons with BPD with versus without PTSD. To review, the 4-D model describes the consciousness of 1) time, 2) thought, 3) body, and 4) emotion in TRASC versus NWC-distress form, and hypothesizes that experiences of TRASC, when compared with NWC-distress, will be: 1) observed less frequently, 2) less intercorrelated, 3) more strongly correlated with other measures of dissociative experiences, and 4) observed more specifically in chronically and developmentally traumatized persons. The theoretical basis for each of these predictions was briefly summarized within the introduction.

Consistent with the above predictions of the 4-D model of trauma-related dissociation (Frewen & Lanius, in press), within our sample of 258 women with BPD, symptoms of TRASC were endorsed less frequently and were more strongly associated with an independent measure of dissociative symptoms (the DES) when compared with symptoms of general distress held by the 4-D model to be part of NWC. These observations replicate prior findings in a PTSD sample (Frewen & Lanius, 2014), shown here to be present in subsamples of BPD patients with and without PTSD comorbidity. As such, the present findings partly serve to support the 4-D model as a transdiagnostic framework, of relevance not only to understanding the symptomatology of PTSD, but to that of other trauma-related disorders including BPD.

However, within the present sample, the 4-D model was only partially supported. First, TRASC symptoms were not found to be less intercorrelated than NWC symptoms on the BSL as was predicted. The latter findings may suggest that, in BPD samples, experiences of dissociation form a more singular dimension or co-occur more often, at least as measured over the past week. Alternatively, this null finding may simply reflect an artifact of use of a psychometric instrument that may be somewhat less fit to measuring the distinctive symptomatology implied by the 4-D model; future research with alternative scales is needed. Indeed a limitation of the present study related to content representation within the BSL for the four symptom dimensions referred to by the 4-D model, wherein only a small number of items were judged by the authors to adequately represent the intended construct.

Second, the relationship between childhood trauma history and TRASC, although generally stronger than that observed for NWC general distress symptoms, and significantly so in the case of emotional neglect within participants with PTSD, was nevertheless, on the whole, not particularly robust. Nevertheless, the present results revealed stronger associations between TRASC and severity of childhood trauma history than was demonstrated in a prior PTSD study which showed little if any relationship other than an association between severity of voice- hearing and magnitude of childhood sexual abuse history (Frewen & Lanius, 2014). Inconsistency of findings across studies could be due to a number of study-level differences including primary diagnosis (BPD vs. PTSD), methods of assessing TRASC, and severity of childhood traumatization; discrepant results therefore await clarification in larger transdiagnostic samples of persons exhibiting a greater range of dissociative symptoms and degree of childhood trauma exposure. Measures of attachment may also be helpful to further elucidate the relationship between adverse childhood experiences and TRASC (e.g., Bureau, Martin, & Lyons-Ruth, 2010; Ogawa, Sroufe, Weinfield, Carlson, & Egeland, 1997). It is further important to note that measures of both TRASC and NWC-distress were significantly correlated with severity of childhood trauma history only in women with BPD comorbid with PTSD. Moreover, women with BPD comorbid with PTSD evidenced a greater level of both dissociation (DES scores) and childhood trauma exposure. Taken together, these findings suggest comorbidity of PTSD in persons with BPD may be a marker for both severity of childhood trauma history and likelihood of the presence of dissociative symptoms, including those expressed as TRASC.

Limitations of the present study include that the four unique symptom dimensions specified by the 4-D model could not be examined separately because of insufficient content representation within the BSL; future studies should administer more comprehensive assessments of TRASC within the BPD population, with the BSL standing only as a surrogate measure of such phenomena in the present sample in the absence of a more adequate measure at the time of data collection. A significant limitation of the present study also involved our reliance on self-report instruments to measure TRASC and NWC-distress; use of objective measures including behavioral and biomarkers of such symptomatology would strengthen confidence in the findings obtained. In addition, sharing the limitation of our prior PTSD study (Frewen & Lanius, 2014), only women were assessed in the present research because sampling was undertaken on data acquired from consecutive admissions to a BPD treatment program for women; whether findings generalize to men requires future investigations.

In addition to addressing the limitations of prior research, future areas of interest include investigating whether the presence of TRASC is significant for predicting outcomes for psychological treatment of both BPD and PTSD as has been shown for dissociative symptoms measured otherwise (Kleindienst et al., 2011; Price et al., 2014). Moreover, given that the nosology of DSM-5 PTSD has been revised, arguably increasing its overlap with BPD (e.g., through inclusion of the symptoms of “pervasive emotional disturbance” and dissociation that are shared with BPD), the transdiagnostic relevance of the 4-D model for PTSD, BPD, and other trauma-related disorders should be investigated for samples diagnosed via current practices. In addition, future studies should investigate associations between the predictions of the 4-D model and hypotheses derived from other theories of trauma-related dissociation, including the structural theory of dissociation (Van der Hart, Nijenjuis, & Steele, 2006). In brief, a major tenet of the structural theory is that persons with dissociative disorders, and in particular those with dissociative identity disorder, experience a sense of self that is compartmentalized, including between one or more facets capable of more or less normal everyday functioning (a so-called apparently normal personality [ANP]), and one or more identity states that evidence a high degree of trauma-related affect dysregulation (a so-called emotional personality [EP]). From the perspective of the 4-D model, whereas an ANP should evidence neither severe NWC-distress nor TRASC, an EP may report experiences of NWC-distress and/or TRASC. As such, the concept of an EP seems independent of those of NWC-distress and TRASC. The structural theory, however, further points out that symptoms of trauma-related dissociation can present in both *positive* form (indicating the presence of a phenomenon not regularly experienced within NWC) and *negative* form (indicating the absence of a phenomenon regularly experienced within NWC); although the notion of positive versus negative symptoms of dissociation is implicit within the conceptual framework described by the 4-D model (e.g., flashbacks and voice-hearing signify positive symptoms, whereas emotional numbing and arguably depersonalization signify negative symptoms), it is not an anchoring principle, and future studies might determine the relative clinical significance of positive versus negative dissociative symptoms in persons with trauma-related disorders. In addition, the structural theory discusses the differentiation between psychoform dissociation (cognitive/affective symptoms) and somatoform dissociation (symptoms of somatization/conversion); the mechanisms underlying manifestations of TRASC of the body in various forms should be compared (e.g., partial and whole-body forms of depersonalization as described by the 4-D model and symptoms of somatization and conversion disorder). Highly relevant to these considerations is a study by Van Dijke and colleagues who found, within a large sample of persons with BPD and/or somatoform disorders, that *inhibitory* symptoms (negative psychoform and somatoform dissociative symptoms and over-regulation of affect) and *excitatory* symptoms (positive psychoform and somatoform dissociative symptoms and under-regulation of affect) are related but distinct clinical phenomena (Van Dijke et al., 2010). In addition, this research group found that only under-regulation of affect was significantly associated with severity of childhood traumatic experiences with caregivers in the same sample (Van Dijke, Ford, Van Son, Frank, & Van der Hart, 2012).

We conclude that the 4-D model offers a novel transdiagnostic framework for investigating the symptomatology of trauma-related disorders as a function of the presence of dissociation, in particular, TRASC. The potential clinical relevance of this framework for informing psychological assessment and treatment including of persons with co-occurring BPD and PTSD requires further study.
